# A phase I trial of SON-1010, a tumor-targeted, interleukin-12-linked, albumin-binding cytokine, shows favorable pharmacokinetics, pharmacodynamics, and safety in healthy volunteers

**DOI:** 10.3389/fimmu.2024.1362775

**Published:** 2024-02-29

**Authors:** Richard T. Kenney, John K. Cini, Susan Dexter, Manuel DaFonseca, Justus Bingham, Isabelle Kuan, Sant P. Chawla, Thomas M. Polasek, Jason Lickliter, Philip J. Ryan

**Affiliations:** ^1^ Sonnet BioTherapeutics, Inc, Princeton, NJ, United States; ^2^ Momentum Metrix, LLC, Dublin, CA, United States; ^3^ Sarcoma Oncology Center, Santa Monica, CA, United States; ^4^ Centre for Medicine Use and Safety, Monash University, Melbourne, VIC, Australia; ^5^ InClin, Inc, San Mateo, CA, United States; ^6^ Nucleus Network Pty Ltd, Melbourne, VIC, Australia

**Keywords:** SON-1010, recombinant IL-12, albumin, fully human albumin binding (FHAB) domain, healthy volunteers, immunotherapy, advanced solid tumors, ovarian cancer

## Abstract

**Background:**

The benefits of recombinant interleukin-12 (rIL-12) as a multifunctional cytokine and potential immunotherapy for cancer have been sought for decades based on its efficacy in multiple mouse models. Unexpected toxicity in the first phase 2 study required careful attention to revised dosing strategies. Despite some signs of efficacy since then, most rIL-12 clinical trials have encountered hurdles such as short terminal elimination half-life (T_½_), limited tumor microenvironment targeting, and substantial systemic toxicity. We developed a strategy to extend the rIL-12 T_½_ that depends on binding albumin *in vivo* to target tumor tissue, using single-chain rIL-12 linked to a fully human albumin binding (F_H_AB) domain (SON-1010). After initiating a dose-escalation trial in patients with cancer (SB101), a randomized, double-blind, placebo-controlled, single-ascending dose (SAD) phase 1 trial in healthy volunteers (SB102) was conducted.

**Methods:**

SB102 (NCT05408572) focused on safety, tolerability, pharmacokinetic (PK), and pharmacodynamic (PD) endpoints. SON-1010 at 50-300 ng/kg or placebo administered subcutaneously on day 1 was studied at a ratio of 6:2, starting with two sentinels; participants were followed through day 29. Safety was reviewed after day 22, before enrolling the next cohort. A non-compartmental analysis of PK was performed and correlations with the PD results were explored, along with a comparison of the SON-1010 PK profile in SB101.

**Results:**

Participants receiving SON-1010 at 100 ng/kg or higher tolerated the injection but generally experienced more treatment-emergent adverse effects (TEAEs) than those receiving the lowest dose. All TEAEs were transient and no other dose relationship was noted. As expected with rIL-12, initial decreases in neutrophils and lymphocytes returned to baseline by days 9-11. PK analysis showed two-compartment elimination in SB102 with mean T_½_ of 104 h, compared with one-compartment elimination in SB101, which correlated with prolonged but controlled and dose-related increases in interferon-gamma (IFNγ). There was no evidence of cytokine release syndrome based on minimal participant symptoms and responses observed with other cytokines.

**Conclusion:**

SON-1010, a novel presentation for rIL-12, was safe and well-tolerated in healthy volunteers up to 300 ng/kg. Its extended half-life leads to a prolonged but controlled IFNγ response, which may be important for tumor control in patients.

**Clinical trial registration:**

https://clinicaltrials.gov/study/NCT05408572, identifier NCT05408572.

## 1 Introduction: Interleukin-12 and SON-1010

First discovered in the late 1980s, Natural Killer Cell Stimulatory Factor, eventually renamed interleukin-12 (IL-12), is a proinflammatory cytokine produced by activated phagocytes and dendritic cells and is a key regulator of cell-mediated immunity (1). Despite the early safety challenges in Phase 2 (2), the clinical development of recombinant human (r)IL-12 and related compounds has been extensive in cancer and immunotherapy indications over the past two decades (3, 4).

As a cytokine, IL-12 has multiple effector functions that bridge the innate and adaptive immune responses in cancer (5) to promote the activation of NK and NKT cells and to polarize CD4^+^ and CD8^+^ T cells. IL-12 has been shown to: a) induce the differentiation of naïve T cells into Th1 cells (6), b) increase the activation and cytotoxic capacities of T and NK cells, c) inhibit the differentiation of Treg cells, and d) inhibit or reprogram immunosuppressive cells such as tumor-associated macrophages (TAMs) and myeloid-derived suppressor cells (MDSCs) (7). IL-12 primarily induces the production of large amounts of interferon gamma (IFNγ), which itself is cytostatic/cytotoxic. Tumor necrosis factor-alpha (TNFα) is also produced by T and NK cells, which reduces the IL-4-mediated suppression of IFNγ (8) and upregulates MHC I and II expression in tumor cells for enhanced recognition and lysis (9, 10). There also appears to be a link between IL-2 and the signal transduction of IL-12 in NK cells. IL-12 stimulates the expression of two IL-12 receptors, IL-12Rβ1 and IL-12Rβ2, maintaining the expression of STAT4, a critical protein involved in IL-12 signaling in NK cells. The enhanced functional response is usually demonstrated by IFNγ production and killing of target cells (11).

IL-12 also exhibits anti-angiogenic activity with increased IFNγ production (12–14), which in turn increases the production of a chemokine called inducible protein-10 (IP-10 or CXCL10) (15). IP-10 then mediates this anti-angiogenic effect. Because of its ability to induce immune responses and anti-angiogenic activity, there has been interest in testing rIL-12 as a possible anti-cancer drug, given its effectiveness in murine tumor models. However, it has not been shown to have substantial activity in many human cancer studies, perhaps due to its toxicity and the short half-life of rIL-12 *in vivo* (16). The potential use of rIL-12 in the treatment of psoriasis and inflammatory bowel disease has also been reported (17).

The antitumor and antimetastatic activities of IL-12 have been extensively demonstrated in murine models, including melanomas, mammary carcinomas, colon carcinoma, renal carcinoma, and sarcomas (18). Studies have addressed the issue of local rIL-12 production versus systemic delivery (i.e., intraperitoneal). Production of rIL-12 at the tumor site (by neoplastic cells engineered to release rIL-12 using appropriate expression vectors) induces rejection of neoplastic cells by CD8^+^ T cells associated with macrophage infiltration, vessel damage, and necrosis (19). Interestingly, the cure rates of mice bearing established tumors were higher after intraperitoneal administration of rIL-12 than after vaccination with tumors releasing rIL-12. Studies using various animal models have expanded our understanding of their potential toxic effects (20). Improved antitumor effects have been observed when rIL-12 is administered along with other cytokines (21) or neoplastic cells expressing costimulatory molecules (22). Analysis of the immune mechanisms activated by IL-12 in these non-clinical models suggested the role of several subsets, including NK cells, CD4^+^ and CD8^+^ T cells, and CD3^+^ CD56^+^ NKT cells expressing the Va14 invariant T-cell receptor (23, 24).

**Figure 1:**
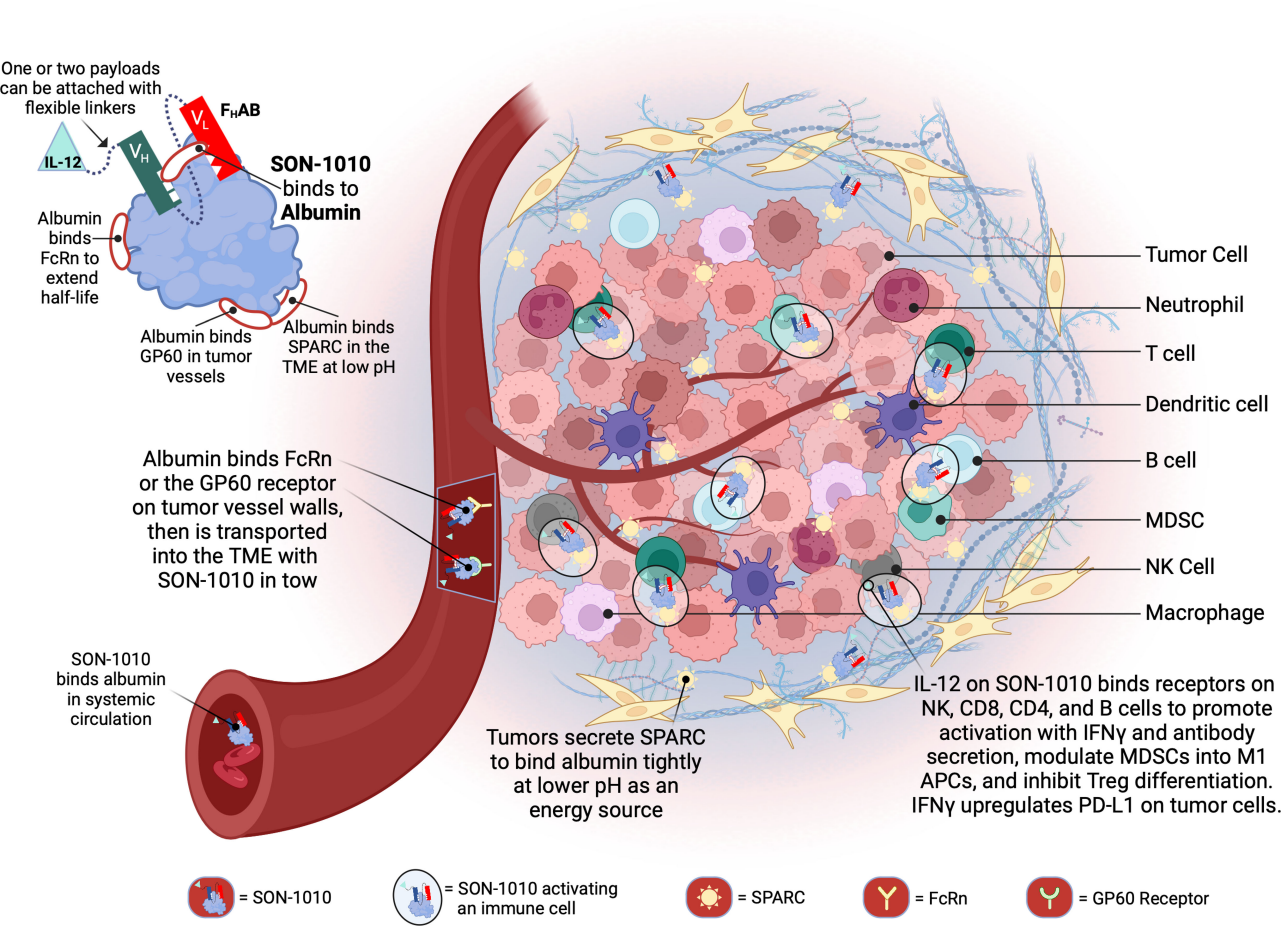
Schematic representation of SON-1010 and its mechanism of action. The F_H_AB portion on the top left consists of a scFv heavy chain (V_H_ in green) linked ([GGGGS]_3_) to a light chain (V_L_ in red) that comprises an albumin-binding domain. Therapeutic payloads can be fused to each side of the central construct using flexible linkers ([GGGGS]_5_). SON-1010 includes single-chain human rIL-12 genetically linked to the F_H_AB V_H_ domain, with no payload or linker added to the V_L_ domain. The scFv binds to a section of albumin at both physiological and acidic pH, without interfering with its binding to FcRn, GP60, or SPARC. Albumin binds systemically to FcRn and SON-1010 can share albumin’s extended PK. The entire complex can be carried into the tumor tissue through the bloodstream, where FcRn and GP60 receptors are upregulated, to be transported across the endothelium into the acidic TME. Once there, albumin binds tightly in dynamic equilibrium via its interaction with SPARC, which is overexpressed in the TME. The rIL-12 cytokine domain can activate resident immune cells and recruit more cells, upregulating the expression of IFNγ from NK, CD4^+^, and CD8^+^ T cells, which then upregulates PD-L1 expression on tumor cells and antibody production from B cells. Created with BioRender.com. FcRn, neonatal Fc receptor; GP60, albondin/glycoprotein 60; PK, pharmacokinetics; SPARC, secreted protein acidic and rich in cysteine; TME, tumor microenvironment.

We designed a proprietary fully human albumin binding (F_H_AB^®^) platform technology (**Figure 1**) that enables the development of innovative targeted biological drugs with enhanced mono- or bi-functional mechanisms (25). SON-1010, the lead drug candidate, is a recombinant, single-chain, unmodified human rIL-12 joined by a flexible ([Gly_4_Ser]_5_) linker to the F_H_AB domain. The platform utilizes a single-chain antibody fragment (scFv) that binds to and “hitch-hikes” on mouse, monkey, or human serum albumin (HSA) for transport to target tissues (26). The initial focus is on immunotherapy of solid tumors; however, the technology is suited for drug development across the spectrum of human diseases, as a number of different domain payloads can be linked to the scFv.

The main limiting factor for the clinical application of rIL-12 monotherapy in solid tumors has been its toxicity and the low level of rIL-12 infiltration and retention in the tumor microenvironment (TME). SON-1010 is being developed as an extended pharmacokinetic (PK) and pharmacodynamic (PD) rIL-12 molecule for the treatment of cancer. The F_H_AB component was designed to enhance the PK of the payload(s) linked to it, which increases the exposure of the side chains to the TME and lymphatic tissue. SON-1010 is carried into the TME because the F_H_AB construct binds to albumin, which then binds to the neonatal fragment crystallizable (Fc) receptor (FcRn) on the surface of endothelial cells, resulting in an increased half-life via cellular recycling of albumin and the F_H_AB that is bound to it (27). FcRn and glycoprotein 60 (GP60) are overexpressed in tumor vessels, promoting the delivery of albumin and its bound IL12-F_H_AB to that space. SON-1010 retention in the acidic TME is facilitated by the albumin complex binding to the “secreted protein acidic and rich in cysteine” (SPARC) protein, which is often expressed in the TME of solid tumors, providing an improved PK profile overall and a dose-sparing effect that decreases toxicity risk, resulting in a broader therapeutic index in mouse models.

Currently, the first-in-human study of SON-1010 (SB101) is being conducted in patients with advanced solid tumors using a multiple ascending dosing (MAD) design (NCT05352750) (28). A second cancer study (SB221) focuses on patients with platinum-resistant ovarian cancer (PROC), in which dose-escalation of SON-1010 is being studied in combination with the anti-PD-L1 immune checkpoint inhibitor atezolizumab in part 1, which will be compared with the standard-of-care in its second part (NCT05756907). In this paper we present the results of SB102, the complementary phase 1 study in healthy volunteers (29), which used a single-ascending dose (SAD) design (NCT05408572), along with preliminary PK/PD results from SB101 for comparison.

## 2 Materials and Methods

### 2.1 Study design

**Figure 2:**
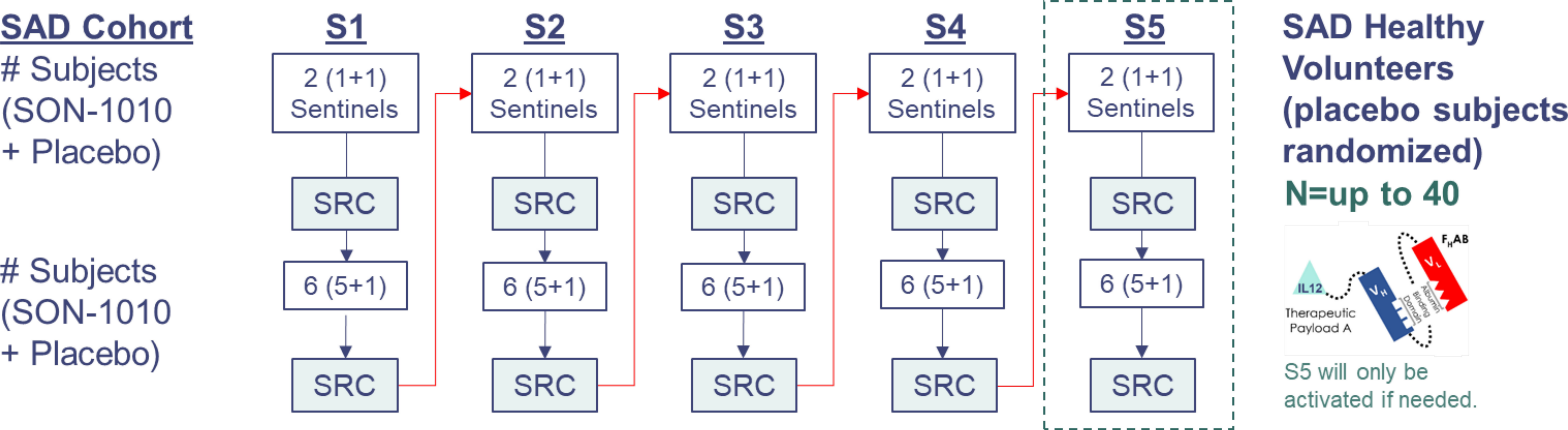
SB102 Trial design. Dosing was organized as cohorts that were to receive 50 ng/kg up to 450 ng/kg (the molar equivalent of 33-300 ng/kg rhIL-12). Safety data from the first four days of sentinel dosing were checked by the SRC before proceeding with the rest of the cohort. Data from days 1 to 22 were reviewed at each SRC cohort meeting. S1, SAD cohort 1; S2, SAD cohort 2; S3, SAD cohort 3; S4, SAD cohort 4; S5, SAD cohort 5; SAD, single ascending dose.

The SB102 study was a phase 1, randomized, double-blind, placebo-controlled, dose-escalation study designed to assess the safety, tolerability, PK, and PD of SON-1010 administered as a single subcutaneous (SC) injection in healthy volunteers (**Figure 2**). A flow diagram for sentinel participants and the rest of each cohort is shown in **Supplementary Figure S1**. Participants had to be 18-54 years old and healthy based on their medical history, physical examination, and clinical laboratory testing (see the full list of Inclusion/Exclusion Criteria in the **Supplementary Material**). SB102 was conducted at a single site in Melbourne, Victoria, Australia; blinding included the participants, site staff, clinical research organization (CRO), sponsor, and medical monitors, as well as the Safety Review Committee (SRC). An exploratory objective was to evaluate the relationship between PK and PD in SON-1010 dosing. Safety was carefully tracked, along with the evaluation of acute inflammatory cytokine responses to help with dose escalation decisions. The study was performed in accordance with the Declaration of Helsinki, Council for International Organizations of Medical Sciences International Ethical Guidelines, and Good Clinical Practice, and was approved by the Alfred Human Research Ethics Committee (Alfred Health, Melbourne, VIC) as authorized by the Australian Government through the National Health and Medical Research Council (NHMRC) on 2Aug2022, once the safety of the first two dose levels had been reviewed in SB101.

A SAD approach was used in SB102, including the dosing of sentinel participants before putting larger numbers at risk, with placebo participants in each of five cohorts designated S1 to S5, with eight participants in each dose cohort randomized to receive either SON-1010 (n=6) or a placebo (n=2) (**Table 1**). Blinding was maintained until the database was closed. One similar trial, a large phase 1 dose escalation study using rIL-12 that was conducted in healthy volunteers as a medical countermeasure for acute radiation syndrome (30), was used as the basis for initial dose selection in the SON-1010 clinical program (31). The first cohort in SB102 received 50 ng/kg SON-1010, which is the molar equivalent of the lowest rIL-12 dose that showed a measurable IFNγ response in that earlier study of healthy volunteers.

**Table 1:** SB102 dose escalation schedule.

Cohort	Planned Number (SON-1010 + Placebo)	Planned Dose (μg/kg)^a^	rIL12 ME (μg/kg)^b^	Actual Number of Participants
**S1**	6 + 2	0.050	0.033	6 + 2
**S2**	6 + 2	0.100 (= 2 x S1)	0.067	6 + 2
**S3**	6 + 2	0.150 (= 1.5 x S2)	0.100	6 + 2
**S4**	6 + 2	0.300 (= 2 x S3)	0.200	5 + 2
**S5**	6 + 2	0.450 (= 1.5 x S4)	0.300	0

a Dose selection was based on all available safety, and real-time pharmacokinetic and pharmacodynamic data. Dosing could change based on prior safety and available PK and PD results.

b ME, Molar equivalent dose of rIL12.

### 2.2 Participant assessment

Participants were followed for 3 days in confinement at the Nucleus Clinical Research Unit and then as outpatients for a total of 28 days after dosing to assess safety, tolerability, and laboratory responses. Safety, including an assessment of adverse events and all available laboratory results, was reviewed at each SRC meeting after all participants in that cohort had completed day 22. If dose-limiting toxicities (DLTs) or a lack of tolerability were observed in at least one participant at a given dose level, the highest previously evaluated dose level without a DLT was defined as the MTD. The cytokine responses are likely to be a better early indicator of an inflammatory response related to SON-1010 than clinical AEs, so, the PD response was followed closely as the dose was escalated with a rapid assessment of ‘acute inflammation’ labs from day to 1-8 (IFNγ, IL1ß, IL-6, IL-8, IL-10, IL-12, and TNFα by a Luminex assay, Crux Biolabs, Melbourne, Australia). The SRC reviewed these results, then authorized the subsequent dose level for enrollment of the next sentinel participants.

Safety and tolerability were determined by assessing treatment-emergent adverse events (TEAEs), vital signs, laboratory test results (hematology, biochemistry, coagulation, thyroid function tests, and urinalysis), electrocardiograms, and physical examination findings. Adverse events were graded according to the current version of the Common Terminology Criteria for Adverse Events (CTCAE) and were categorized as mild (grade 1), moderate (grade 2), severe (grade 3), life-threatening (grade 4), or death (grade 5), or as serious AEs (SAEs) according to standard definitions. Causal relationships of TEAEs to SON-1010 administration were judged by the investigator as either “unrelated”; or “possibly”, “probably”, or “definitely” related (treatment-related TEAEs). All TEAEs were coded using the Medical Dictionary of Regulatory Activities (MedRA v24.0).

Serum SON-1010 concentrations were assessed using a validated electrochemiluminescence-based immunoassay (ECLIA) for IL-12 (Mesoscale Discovery [MSD] Cat# K151QVD) at Celerion (Omaha, NB, USA). Urine concentrations were evaluated at 360biolabs (Melbourne, Australia) with the same kit after qualification for its use with urine. Primary PK parameters were calculated from concentration versus time data using non-compartmental analysis (NCA), including maximum serum concentration (C_max_); area under the serum concentration vs. time curve (AUC) from the first dose until 24, 48, or 168 h (AUC_0-x_); AUC until the last quantifiable concentration (AUC_0-t_); an estimate of the total AUC (AUC_inf_); time to peak serum concentration (T_max_); terminal elimination half-life (T_½_), apparent clearance (CL/F); and apparent volume of distribution (V_z_/F). The formal PD parameters included IFNγ, IL-1β, IL-2, IL-4, IL-6, IL-8, IL-10, and TNFα levels, which were determined using a qualified multiplex assay (MSD Cat# K15049G) (Celerion).

### 2.3 Statistics

The analysis sets for this study included the enrolled set (participants who signed the informed consent form [ICF], met eligibility criteria, and were approved for randomized treatment), the safety set (all participants who received at least one dose of SON-1010 or placebo), and the PD set (participants with sufficient PD samples available). All analysis datasets and outputs were produced by the Biostatistics Department of Resolutum Global using SAS^®^ Version 9.4 (SAS Institute, Cary, North Carolina, USA). The sample size selected (6 active participants per cohort) was based on common practice in phase 1 dose escalation studies. Non-compartmental analysis to estimate PK parameters was performed using R version 4.3.0 (32) and the pkr package version 0.1.3 (R Foundation for Statistical Computing, Vienna, Austria).

## 3 Results

SON-1010 is composed of a F_H_AB domain that is genetically linked to the N-terminus, using a short, non-immunogenic amino acid repeat sequence designed to avoid steric hindrance ([Gly_4_Ser]_5_), to single-chain rIL-12. The molecule binds albumin in the serum after injection to share its extended PK, and the complex is distributed to the tumor tissue after binding to FcRn or GP60 (27). The complex also binds SPARC avidly at a lower pH (26), which is often found in the TME, where the cytokine can then interact with resident immune cells (**Figure 1**). The SB102 trial evaluated four single-dose cohorts of healthy volunteers given SON-1010 at 50, 100, 150, or 300 ng/kg, or placebo (**Table 1**), and was designed to support the SB101 MAD study in patients with cancer (29, 33). The planned fifth cohort of SB102 was not enrolled, to avoid potential adverse events at higher doses (**Figure 2**). The maximal dose in this study will be used as the ‘desensitizing first dose’ in cancer patients to take advantage of the known rIL-12 tachyphylaxis and controlled increases in IFNγ (2, 4, 34), so a higher MTD can be targeted with subsequent maintenance doses.

The median age of the 31 participants in the study population (23 active, 8 placebo) was 28.0 years (range: 18.0 to 52.0 years). Twenty-one participants (67.7%) were male. Of the 10 female participants, all but one was of child-bearing potential. Most (19/31, 61.3%) participants were Caucasian or Asian (11/31, 35.5%), and none were Hispanic or Latino. The median body mass index (BMI) was 24.10 kg/m^2^ (range: 19.4 to 30.7 kg/m^2^). This profile was consistent across participants receiving SON-1010 or placebo and across SON-1010 dose cohorts.

The participants were required to be generally healthy to be enrolled in the study. The most frequently reported (≥10%) medical and surgical histories pertained to procedures or infections (12/31, 38.7% each); eye disorders (10/31, 32.3%); injuries and procedural complications (8/31, 25.8%); psychiatric disorders (6/31, 19.4%); and respiratory, mediastinal, and thoracic disorders or skin and subcutaneous tissue disorders (5/31, 16.1% each). The most frequently reported historical conditions were COVID-19 (9/31, 29.0%), myopia (7/31, 22.6%), wisdom tooth removal (5/31, 16.1%), and tonsillectomy, astigmatism, or depression (4/31, 12.9% each).

### 3.1 Safety and tolerability in healthy volunteers

Blinded dosing in each cohort started with two sentinel participants (one active and one placebo), followed approximately a week later by six participants in the rest of each cohort to assess the safety, tolerability, PK, and PD without the background of prior chemotherapy (29). SON-1010 administration was generally safe and well-tolerated at all doses in this population of healthy volunteers. There were no grade ≥ 3 treatment-emergent adverse events (TEAEs) that were considered related to treatment. There were no serious adverse events (SAEs) and no TEAEs leading to discontinuation of the study.

Participants receiving SON-1010 at doses of 100 ng/kg or higher tolerated the injection but generally experienced more TEAEs than participants receiving SON-1010 at 50 ng/kg (**Table 2**). However, there was no clear evidence of a dose-related effect among the higher-dosing cohorts. Headache (10/23, 43.4%), myalgia, injection site pain or induration, and pyrexia (3/23, 13.0% each) were reported as related events more frequently among SON-1010 treated participants than among placebo group, who only included one with headache or injection site pain (1/8, 12.5%), with no clear relationship between the SON-1010 dose cohort and frequency. Most TEAEs were mild and were considered possibly, probably, or definitely related to SON-1010. One participant (12.5%) in Cohort S1 developed grade 2 neutropenia on day 5 that returned to normal by day 10. One participant each (12.5%) in Cohorts S2 and S3 reported a recurrent moderate/grade 2 headache requiring acetaminophen for control, and one participant (14.3%) in Cohort 4 reported grade 2 flu-like symptoms requiring acetaminophen for 5 days. One placebo participant (12.5%) reported a grade 2 headache requiring acetaminophen that was considered related as well. During the trial, the most frequently prescribed concomitant medication was acetaminophen (15/31, 48.4%), which was administered to one placebo vs. five of the six active participants in the SON-1010 100 ng/kg cohort. Other concomitant medications were administered to a single participant, with no apparent patterns across treatments or cohorts.

**Table 2:** SB102 adverse events considered related to SON-1010.

Preferred Term (PT)^a^	50 ng/kg (N=6) n (%)	100 ng/kg (N=6) n (%)	150 ng/kg (N=6) n (%)	300 ng/kg (N=5) n (%)	Placebo (N=8) n (%)
**Injection site reaction**				2 (40.0%)	
**Injection site erythema**		1 (16.7%)	1 (16.7%)		
**Injection site pain**		2 (33.3%)	1 (16.7%)		1 (12.5%)
**Injection site induration**		1 (16.7%)	2 (33.3%)		
**Pyrexia**			1 (16.7%)	2 (40.0%)	
**Axillary pain**			2 (33.3%)		
**Fatigue**		1 (16.7%)			1 (12.5%)
**Chills**		1 (16.7%)			
**Malaise**			1 (16.7%)		
**Headache**	1 (16.7%)	3 (50.0%)	2 (33.3%)	2 (40.0%)	
**Dizziness**		1 (16.7%)			
**Somnolence**	1 (16.7%)				
**Myalgia**	1 (16.7%)		1 (16.7%)	1 (20.0%)	
**Neck pain**		1 (16.7%)			
**Abdominal pain**			1 (16.7%)		1 (12.5%)
**Diarrhea**		1 (16.7%)			
**Nausea**					1 (12.5%)
**Neutropenia**		1 (16.7%)			
**Iron deficiency anemia**		1 (16.7%)			
**Hordeolum**					1 (12.5%)
**Upper Respiratory Infection**		1 (16.7%)			
**Night sweats**			1 (16.7%)		
**Rash**			1 (16.7%)		
**Transaminases increased**				1 (20.0%)	
**Blepharospasm**	1 (16.7%)				
**Hot flush**					1 (12.5%)
**Influenza like illness (Grade 2)**				1 (20.0%)	
**Headache (Grade 2)**		1 (16.7%)	1 (16.7%)		1 (12.5%)
**Neutropenia (Grade 2)**	1 (16.7%)				

a All TEAEs considered to be possibly, probably, or definitely related to the injection were grade 1, apart from those noted as grade 2. N = number in group; n = number with event.

**Figure 3:**
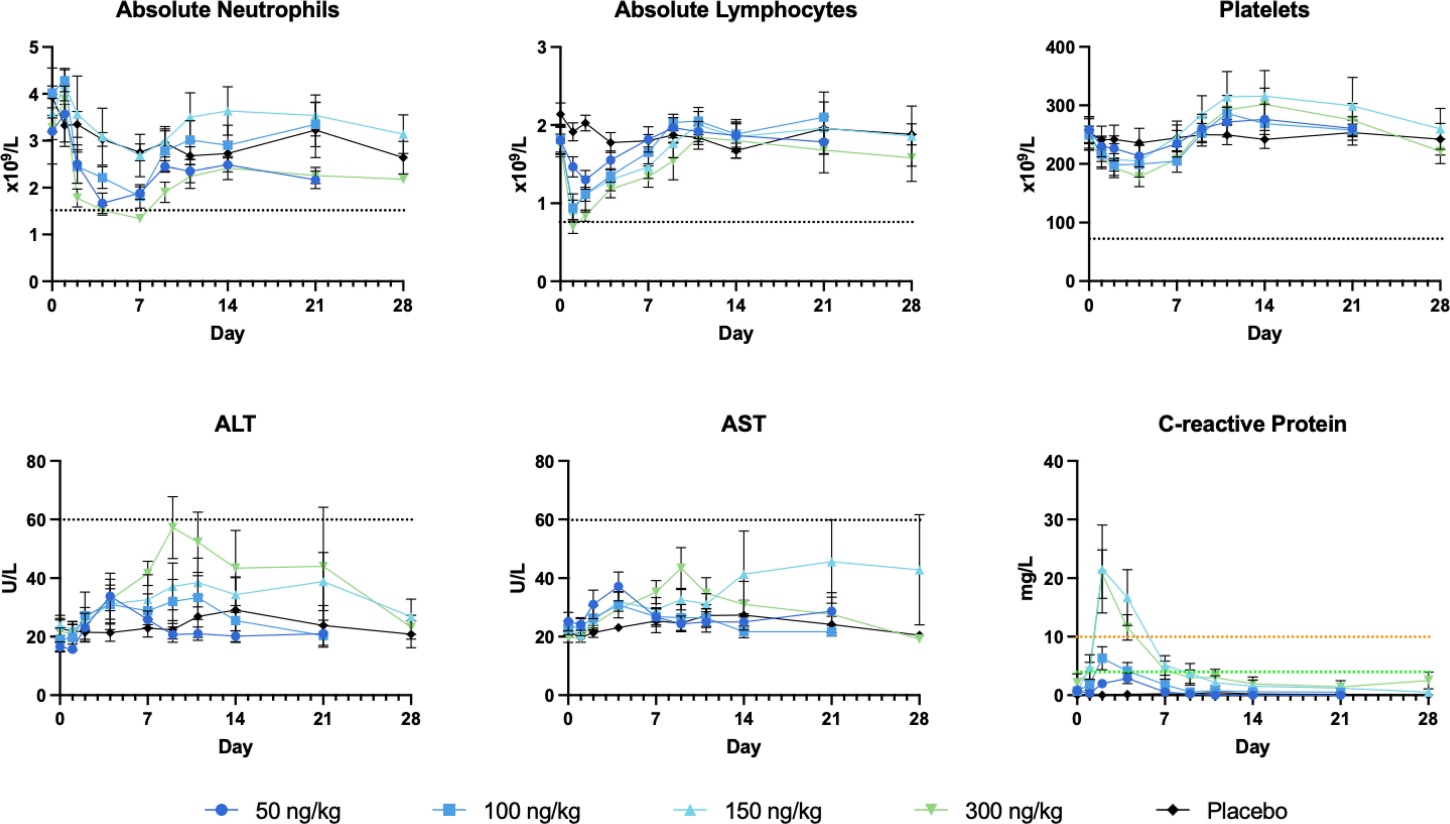
Safety laboratory results. The most reactive laboratory results are shown in each panel, along with the grade 1 limit (35) as a black dotted line. C-reactive protein has no defined AE limits: normal < 3 mg/L, minor increase 3-10 mg/L, moderate increase 10-100 mg/L. ALT, alanine aminotransferase, AST, aspartate aminotransferase.

Dose-related decreases in neutrophils, lymphocytes, and platelets were observed 24-72 hours after the administration of SON-1010 (**Figure 3**), with resolution by day 7 to 10, which is consistent with other studies that used rIL-12 (30) or rIFNγ (36). Alanine aminotransferase (ALT) levels increased over the first 5 to 10 days then returned to baseline, with lower increases in aspartate aminotransferase (AST) levels or other liver enzymes. All values returned to baseline within a short period of time. A dose-related increase in C-reactive protein concentration was also observed on day 2, which returned to baseline values by day 7. Acute inflammation was assessed in each cohort by Luminex assay, for review by the SRC (**Supplementary Figure S2**). An increase in IFNγ was observed in all SON-1010 dose cohorts, as well as a much smaller dose-dependent increase in TNFα, IL-8, and IL-10 concentrations. These changes occurred within 24 to 48 h after administration of the study drug. There were minimal transient increases in IL-6 concentrations that were not dose related. All values returned to baseline within a few days. There were no notable changes in vital signs or electrocardiograms. No TEAEs or cytokine responses were observed that might suggest cytokine release syndrome (37).

### 3.2 Pharmacokinetics

**Figure 4:**
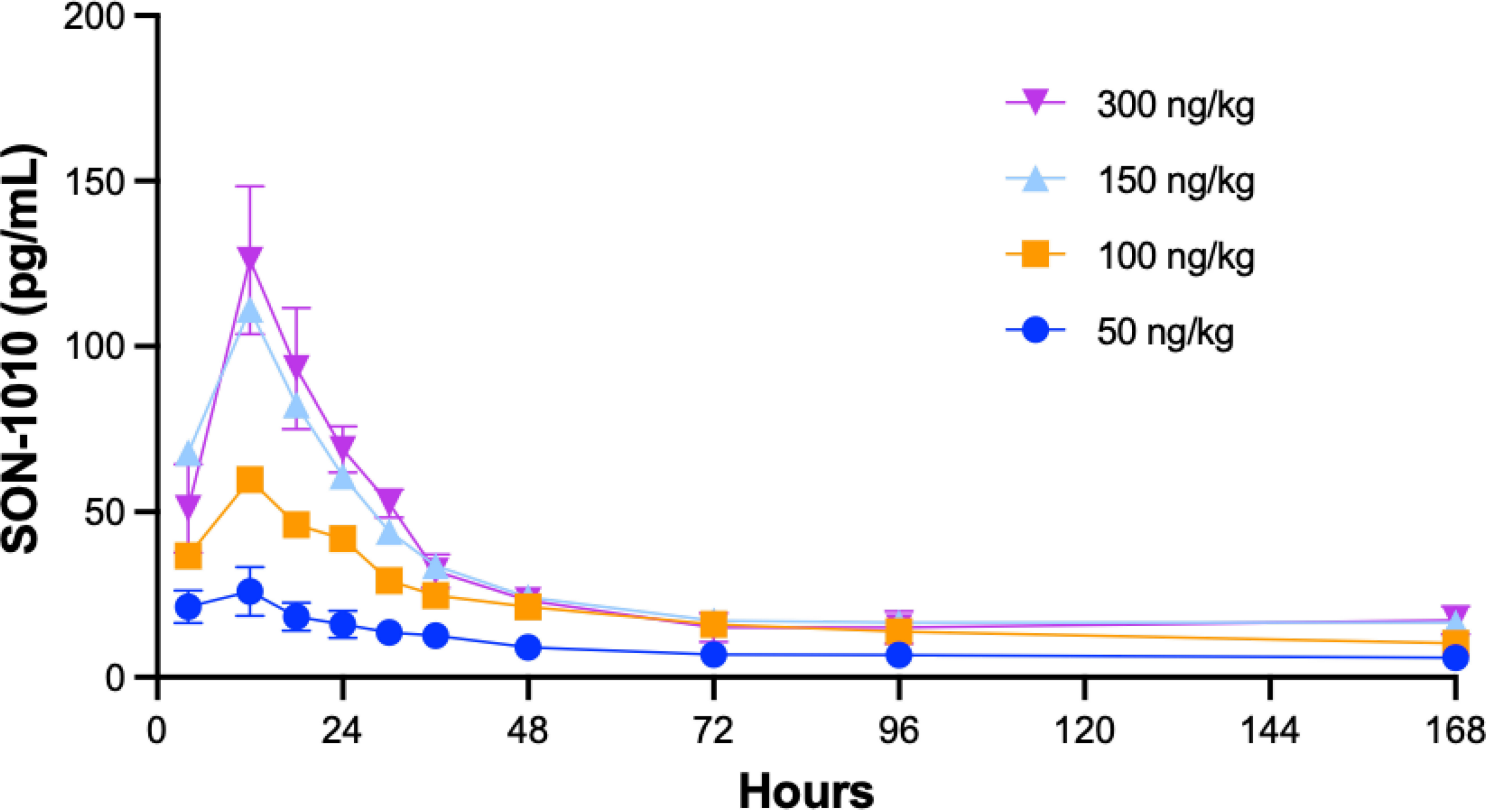
SON-1010 Concentration over time by SB102 cohort. Healthy volunteers in study SB102 were given a single dose of SON-1010 SC and followed closely for safety, PK, and PD over the course of 4 weeks. The geometric mean levels of SON-1010 are shown with error bars (geometric mean CV%) for the lowest and highest groups.

Studying SON-1010 in healthy volunteers in SB102 was an important objective for this non-genotoxic therapeutic oncology drug candidate, as it provided an opportunity to evaluate PK and PD without interference from prior chemotherapy (29, 33). Mean serum concentration versus time profiles following the single SC injection of SON-1010 are presented for the first week (**Figure 4**). Between the SON-1010 lowest- (50 ng/kg) and highest- (300 ng/kg) dose cohorts (a 6x escalation in dose), the serum C_max_ increased by 4.5x (Momentum Metrix, Dublin, CA), and the time to reach that (T_max_) was approximately 11 h (**Table 3**). This was associated with a corresponding 4.5 × increase in the exposure area under the concentration time curve (AUC) from time zero to the time of last observable concentration (AUC_0-t_), and the shape of the curves indicated typical two-compartment elimination kinetics (**Figure 4**). The mean T_½_ across all dose cohorts was 104 h, and the serum concentrations for the majority of the participants remained above the lower limit of quantitation (LLOQ) for 336 h. The mean C_max_ value increased in a less than proportional manner between dose cohorts, yielding nonlinear PK. The geometric mean C_max_ values of the low- and high-dose cohorts were 29 pg/mL and 131 pg/mL, respectively. The low dose cohort reported mean AUC_0-t_ and AUC_0-inf_ of 1340 (CV% 41.5) h•pg/mL and 1500 (CV% 8.5) h•pg/mL, respectively. The high-dose cohort reported 6030 (CV% 47.1) h•pg/mL and 9850 h•pg/mL, respectively. Urine SON-1010 concentrations were below the level of quantitation at all time points, so that route of elimination was not included in the analysis. The SON-1010 PK parameters C_max_, AUC_0-24_, and AUC_0-48_, are shown graphically in **Supplementary Figure S3A**. There was relatively large variability in the mean SON-1010 PK parameters with poor linear fits (R^2^ adj < 0.8), and nearly all parameters had a geometric mean CV% greater than 30%; therefore, N was too low to calculate accurate summary statistics for other PK parameters, such as CL/F and V_Z_/F.

**Table 3:** Pharmacokinetic summary statistics by dose cohort.

Cohort	Statistic	C_MAX_ (pg/mL)	T_MAX_ (h)	AUC_0-24h_ (h*pg/mL)	AUC_0-48h_ (h*pg/mL)	AUC_0-t_ (h*pg/mL)	AUC_0-inf_ (h*pg/mL)	T_1/2_ (h)
**S1: 50 ng/kg** (N=6/5M/1F)	GM	29.3	9.80	454	772	1,340	1,500	69.1
	CV%	71.7	34.9	70.7	52.4	41.5	8.5	159.2
**S2: 100 ng/kg** (N=6/3M/3F)	GM	68.2	11.0	1,110	1,820	3,610	5,370	138
	CV%	104.3	66.2	100.5	72.6	39.6	32.8	50.9
**S3: 150 ng/kg** (N=6/4M/2F)	GM	125	11.2	1,970	2,930	5,570	10,200	112
	CV%	40.6	31.2	38.8	40.8	58.7	22.2	34.7
**S4: 300 ng/kg** (N=5/4M/1F)	GM	131	11.1	2,050	3,050	6,030	9,850	110
	CV%	39.4	18.3	36.2	23.7	47.1	NA	NA

AUC, area under the serum concentration vs time curve; AUC_0-x_, AUC from the first dose until the time indicated or (t) the last quantifiable concentration; C_max_, maximum observed serum concentration; CV%, geometric mean coefficient of variation; GM, geometric mean; h, hours; NA, not applicable; T_max_, time to peak serum concentration; T_½_, terminal elimination half-life.

**Figure 5:**
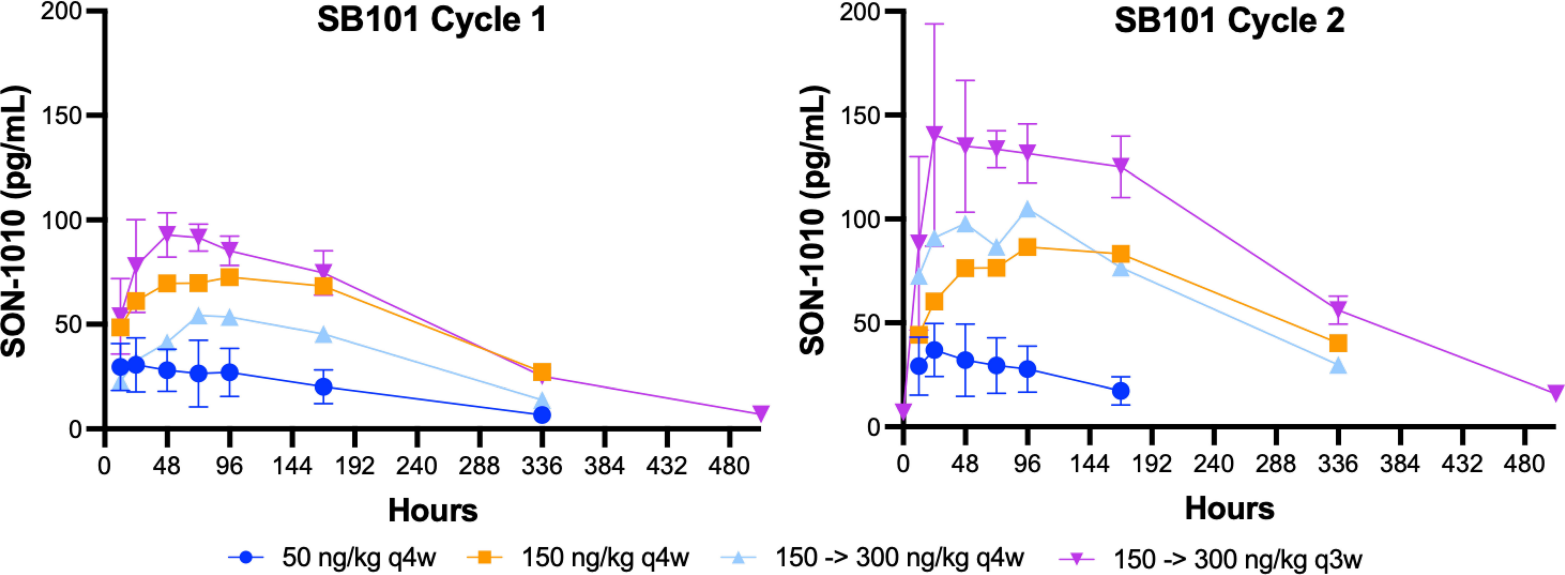
SON-1010 concentration over time by SB101 cohort. Patients in study SB101 were administered a fixed dose of SON-1010 (in the first two groups) or a desensitizing first dose followed by a higher maintenance dose (in the last two groups). The dose interval was reduced from every 4 weeks (q4w) to q3w in the last (and subsequent) groups. Error bars (geometric mean CV%) are shown for the lowest and highest groups, respectively.

Interim data from repeat dosing in patients with advanced solid tumors in study SB101, including dose escalation up to the same maximum dose used in SB102, are now available (**Figure 5**) (28). Interestingly, the SON-1010 concentration curves, using the same assay in cancer patients, compared with a single dose in healthy volunteers (**Figure 4**) showed an atypically dissimilar contour. Single-compartment elimination kinetics were noted in patients with cancer, compared to the two-compartment elimination kinetics observed in the healthy volunteers. The C_max_ and AUC PK parameters in SB101 were similar after the second dose compared to the first dose in SB102, while the IFNγ PD parameters of C_max_ and AUC were suppressed in SB101 (**Supplementary Figures S3B, S4B**), presumably by the induction of SOCS proteins (34).

### 3.3 Pharmacodynamics

**Figure 6:**
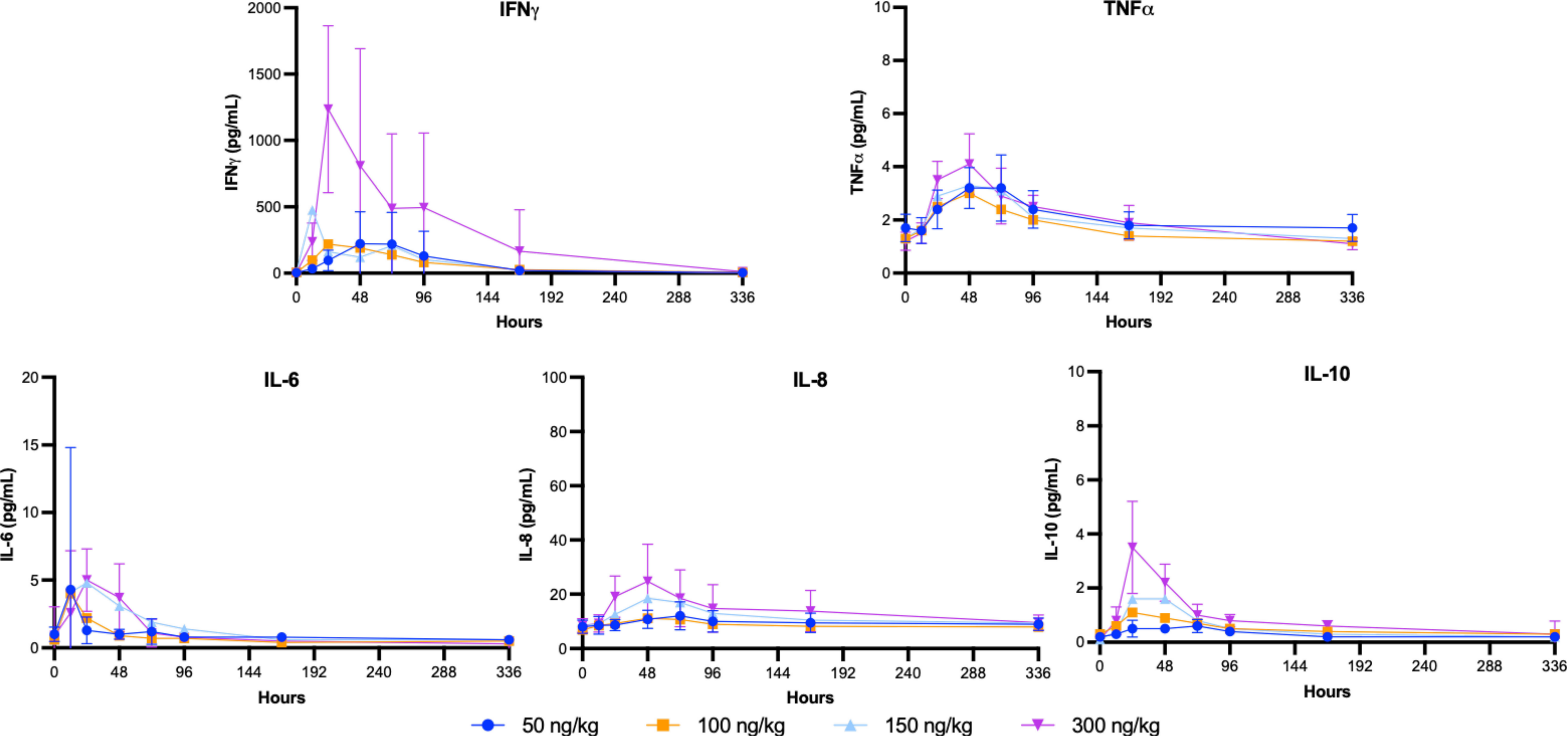
SB102 Cytokine concentrations over time. Serum was collected over the course of the study for PD analysis to correlate with the PK findings. The geometric mean levels of each cytokine are shown with error bars (geometric mean CV%) for the lowest and highest groups.

Endogenous biomarkers of interest included IFNγ, IL-1β, IL-2, IL-4, IL-6, IL-8, IL-10 and TNFα. Of these, only IFNγ, TNFα, IL-6, IL-8, and IL-10 met the criteria for analysis, as fewer than 20% of the data were below the limit of quantitation. Mean serum concentration versus time profiles following the single SC injection of SON-1010 are presented for the first 2 weeks (**Figure 6**). Apart from IL-6, the concentrations remained above the LLOQ for all study participants.

A summary of the NCA parameter values after a single SC dose showed that IFNγ was the most prominent cytokine responding (**Table 4**). The mean C_max_ value disproportionately increased between the wide range of doses tested, peaking at 977 pg/mL in the highest dose cohort (300 ng/kg). The time taken to achieve maximal IFNγ blood concentrations varied greatly between cohorts and did not correlate with the dose, with the mean time required to peak ranging from 28.8 to 85.0 hours. The AUC_0-t_ also increased disproportionately following the cohort doses and rose to 106,000 h*pg/mL in the highest-dose cohort. However, the partial areas under the concentration-time curve from time zero to 24 h, 48 h, and 168 h increased in a dose-dependent manner. The SON-1010 PD parameters C_max_, AUC_0-24_, and AUC_0-48_, are shown graphically in **Supplementary Figure S4A**.

**Table 4:** Pharmacodynamic summary statistics by dose cohort.

Cohort	Analyte	Statistic	C_MAX_ (pg/mL)	T_MAX_ (h)	AUC_0-24h_ (h*pg/mL)	AUC_0-48h_ (h*pg/mL)	AUC_0-168h_ (h*pg/mL)	AUC_0-t_ (h*pg/mL)
**S1: 50 ng/kg** (N=6/5M/1F)	IFNγ	GM	342	85.0	1,030	5,190	21,100	34,200
		CV%	64.4	137.6	65.9	71.4	92.9	78.0
	IL-10	GM	0.8	67.7	8.3	21.5	78.7	135
		CV%	28.5	187.6	62.7	50.6	40.2	129.4
	IL-6	GM	4.9	16.2	69.4	110	238	740
		CV%	157.6	83.6	168.3	92.3	50.7	88.4
	IL-8	GM	13.0	72.2	205	440	1,700	6,240
		CV%	38.7	48.0	31.0	28.5	33.5	29.1
	TNFα	GM	3.4	54.9	44.0	112	409	1,270
		CV%	26.8	21.1	29.3	26.5	27.8	24.2
**S2: 100 ng/kg** (N=6/3M/3F)	IFNγ	GM	331	30.3	2,630	8,730	21,800	27,600
		CV%	65.3	61.5	142.6	74.4	28.6	20.9
	IL-10	GM	1.4	38.9	16.1	41.6	113	262
		CV%	63.8	59.2	71.4	62.0	51.1	29.6
	IL-6	GM	4.2	15.2	67.1	107	191	212
		CV%	42.1	36.6	36.2	39.9	23.1	84.7
	IL-8	GM	12.7	54.8	200	449	1,590	5,760
		CV%	33.2	20.7	16.8	20.9	20.8	20.3
	TNFα	GM	3.1	45.7	42.7	109	352	981
		CV%	13.1	36.3	22.3	20.7	12.9	15.7
**S3: 150 ng/kg** (N=6/4M/2F)	IFNγ	GM	573	28.8	4,070	12,500	32,800	41,800
		CV%	66.3	68.9	160.5	126.5	62.8	68.7
	IL-10	GM	2.1	38.1	15.4	57.3	138	236
		CV%	88.9	36.9	107.2	83.0	65.0	90.2
	IL-6	GM	5.2	24.0	82.4	184	373	587
		CV%	55.0	46.0	67.1	58.2	43.3	47.2
	IL-8	GM	20.3	48.8	238	617	2,280	7,630
		CV%	64.9	41.4	50.4	57.2	47.6	64.2
	TNFα	GM	3.8	43.5	46.2	123	407	1,130
		CV%	35.1	52.4	34.5	32.5	25.0	33.2
**S4: 300 ng/kg** (N=5/4M/1F)	IFNγ	GM	977	52.3	7,230	26,700	86,000	106,000
		CV%	91.7	55.4	59.2	83.8	97.9	105.4
	IL-10	GM	2.75	36.4	21.6	75.9	195	352
		CV%	51.3	39.4	64.2	52.0	32.3	73.1
	IL-6	GM	5.4	27.6	66.3	150	259	413
		CV%	80.5	63.1	84.9	44.6	32.0	50.5
	IL-8	GM	24.6	52.2	246	718	2,620	7,100
		CV%	53.3	18.6	29.0	42.4	50.8	36.4
	TNFα	GM	4.6	48.1	40.3	135	445	993
		CV%	27.8	0.2	19.1	25.2	23.6	19.4

AUC, area under the serum concentration vs time curve; AUC_0-t_, AUC from the first dose until the last quantifiable concentration; C_max_, maximum observed serum concentration; CV%, geometric mean coefficient of variation; GM, geometric mean; h, hours; T_max_, time to peak serum concentration.

Another consideration is to compare SON-1010 PK with the IFNγ PD response after a single dose in healthy volunteers. The greatest linear correlation was observed between C_max_ PK and AUC_0-24h_ IFNγ PD (Pearson correlation coefficient = 0.77, *p*<0.001) (**Supplementary Figure S5**). Although it was much less responsive, the C_max_ value for IL-10 increased with each higher SON-1010 dose cohort, peaking at 2.75 pg/mL with the highest dose. The mean time taken to achieve C_max_ ranged from 36.4 to 67.7 h. Although the analyzed IL-10 AUC metrics appeared to suggest dose proportionality, this was less clear for the other cytokines studied. The mean maximum IL-6, IL-8, and TNFα concentrations achieved after a 300 ng/kg dose of SON-1010 were 5.4, 24.6, and 4.6 pg/mL, respectively. The mean times to achieve C_max_ were 27.6, 52.2, and 48.1 h, respectively.

## 4 Discussion

### 4.1 Development of rIL-12

Early efforts to advance rIL-12 into the clinic showed in the first phase 1 study that doses up to 500 ng/kg daily could be administered intravenously (IV) with acceptable levels of safety, starting two weeks after a test dose (4). Weekly SC dosing was also well-tolerated at that dose (38). However, in the subsequent phase 2 study of 17 patients who received daily rIL-12 IV, 12 patients were hospitalized and two patients died (2). A thorough scientific investigation to determine the cause of this unexpected toxicity failed to identify any difference in the drug products used or the patient populations enrolled in the two IV studies that could have accounted for the profound difference in toxicity. The schedule-dependent toxicity of rIL-12 and an abrupt increase in IFNγ levels were verified in mice and nonhuman primates to be a form of tachyphylaxis; therefore, the dosing level misdirection was thought to have been due to PD effects.

**Figure 7:**
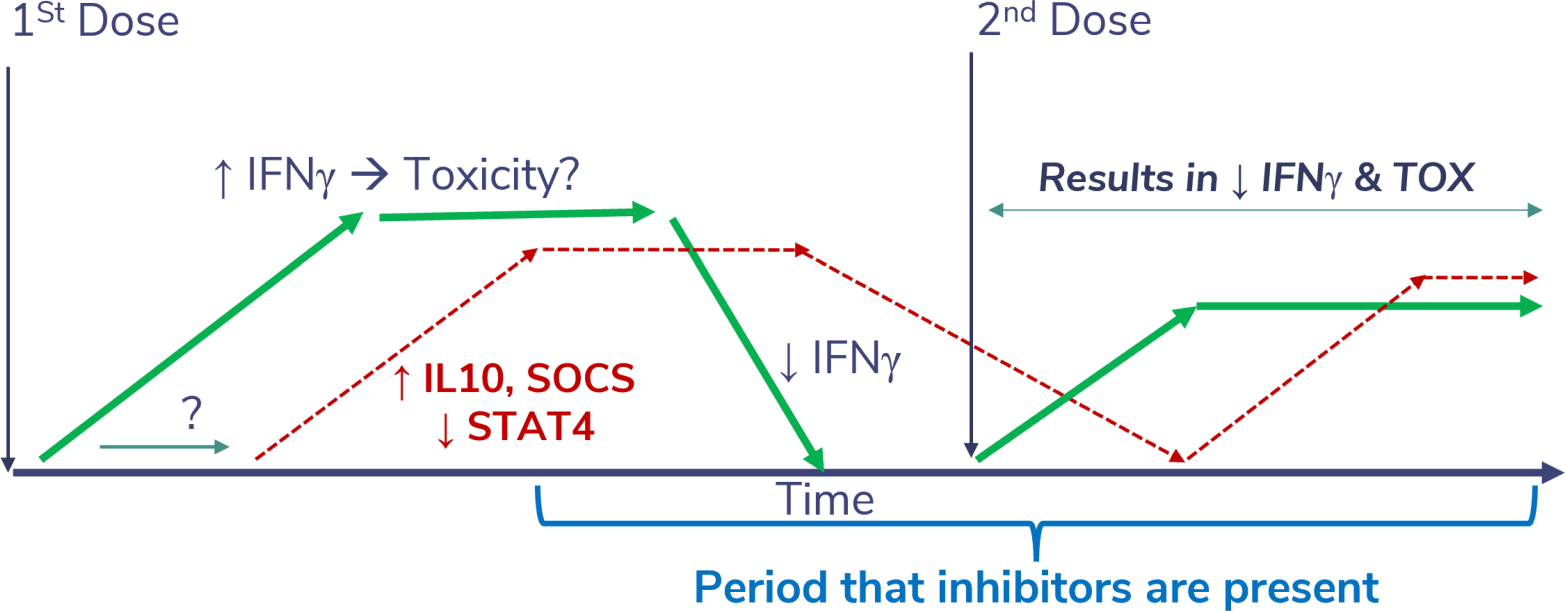
Theoretical impact of a desensitizing dose of SON-1010. Injection of rIL-12 leads to a sustained increase in IFNγ levels, which can cause significant toxicity if it reaches much higher levels acutely. This appears to be physiologically limited by SOCS proteins (34). If a second dose of rIL-12 or SON-1010 is given while SOCS is still inhibiting the phosphorylation of STAT4, the resulting toxicity potential should be abrogated, possibly allowing for a higher MTD.

The single test dose injection of rIL-12 administered 2 weeks before consecutive dosing in that first phase 1 study, but not after daily administration in the phase 2 study, apparently had a profound abrogating effect on rIL-12-induced IFNγ production and toxicity with subsequent doses. This was likely a result of the induction of suppressors of cytokine signaling (SOCS) proteins (**Figure 7**) that normally regulate inflammatory responses (34). Competitive binding of SOCS proteins to the phosphorylated tyrosine residues of cytokine receptors prevents the docking of STAT proteins and the transfer of the signals, thereby reducing toxicity. When the second rIL-12 dose was delayed in phase 1, the subsequent toxic effects may have been suppressed by lingering SOCs proteins. However, in the next study the mean serum IFNγ levels rose to over 25,000 pg/mL after starting with daily injection of rIL-12. This was attributed, at least in part, as the cause of the toxicity, compared to an IFNγ peak of 5,000 pg/mL in phase 1 after the test dose. Interestingly, the phase 2 IFNγ peak was about the same peak level that was measured after intramuscular administration of rIFNγ at its MTD of 5.0 mg/m^2^ in patients with cancer in an early phase 1 trial (36), where the most notable toxicity was fatigue. Peak IFNγ levels appeared to correlate with maximum toxicity in that study. Perhaps the more severe toxicity observed in the phase 2 study of rIL-12 was related to the sustained level of IFNγ that was secondarily induced, which had peaked at approximately 10,000 pg/mL after IV administration of 500 ng/kg rIL-12 in phase 1 (4).

Since the initial phase 2 study of rIL-12 (2), numerous trials have been conducted to determine the optimal dosing schedule and potential utility of various forms of rIL-12, both in patients with cancer and in healthy volunteers. In the largest study to date of rIL-12 in healthy volunteers, designed to study its use as a medical countermeasure for humans exposed to lethal radiation, 32 subjects were initially enrolled in a SAD format starting at 2 μg (as a standardized dose) and the MTD was found to be 12 μg when given SC as a standardized dose (30). The maximum serum concentration (C_max_) values generally increased with increasing dose levels, except for the 20 μg cohort, where only one participant was dosed. Sixty patients were enrolled in a placebo-controlled expansion study at the MTD of 12 μg. In both studies, the most common AEs related to rIL-12 were headache, dizziness, and chills during the first few days of treatment. No immunogenicity was observed. Two-compartment elimination of rIL-12 was noted, suggesting significant distribution into extravascular spaces; the initial T_½_ was 8.7 ± 4.7 hours and the C_max_ was 57.7 ± 49.8 pg/mL. rIL-12 triggered transient reductions in neutrophils, platelets, reticulocytes, lymphocytes, NK cells, and CD34^+^ hematopoietic progenitor cells by day 2, and correlated with induced increases in IFNγ and CXCL10. The cell subsets returned to above normal by day 7, and all parameters normalized over 1–2 weeks. This suggests that splenic sequestration or margination may account for the cell count changes, rather than destruction, as the return to normal in the standard complete blood count was faster than would be expected from simple replacement.

Multiple studies in patients with various types of cancer have shown similar effects of rIL-12 dosing strategies, ranging from daily to 2 to 3 times weekly. Most studies reported an MTD of 500–1000 ng/kg after IV or SC administration. Several attempts have been made to extend T_½_ for a superior PK profile; the most advanced is NHS-IL12, an immunocytokine composed of two rIL-12 heterodimers, each fused to the heavy chain of an antibody that binds to DNA (39). The C_max_ of NHS-IL12 was reached at 36 h, and time-dependent elevations of IFNγ and IL-10 were observed after SC NHS-IL12 administration, which returned to normal by day 7. The MTD of NHS-IL12 was determined to be 16.8 μg/kg, which is much higher than the MTD for rIL-12 of 0.5 to 1 μg/kg, suggesting the possibility of steric hindrance of the cytokine portion of NHS-IL12. Note that NHS-IL12 was originally administered every 4 weeks as monotherapy and is currently being developed in combination with a checkpoint inhibitor given every 2 weeks (40).

### 4.2 Evaluation of SON-1010

The preclinical testing of SON-1010 has been extensive (25, 41). An early proof-of-concept study of F_H_AB in the TGFβ^+^ mouse 4T1 breast tumor model (42) showed the accumulation and prolonged retention of F_H_AB in the tumor as well as when anti-TGFβ was linked to the scFv, whereas the anti-TGFβ antibody alone first accumulated in tumor tissue, then rapidly diffused out. In another study, murine (m) rIL-12 linked to F_H_AB caused up to a 10-fold increase in serum half-life in mice, compared with the rIL-12 control (41).

Murine rIL-12 (mIL-12) causing a reduction in pulmonary metastases or SC growth of B16F10 melanoma in mice was demonstrated as early as 1993 (23). Dose-dependent increases in anti-tumor activity were also demonstrated with mIL12-F_H_AB in that melanoma model, producing a corresponding increase in tumor-infiltrating activated NK and CD8^+^ T cells (41). Single doses of mIL12-F_H_AB were up to 30-fold more effective (by molar equivalence) in reducing B16F10 tumor growth and extending survivability, compared with mIL-12 alone, in a dose-dependent manner in tumor-bearing mice compared to placebo. This resulted in a corresponding increase in the immune response, as reflected by the increased splenic weight and serum IFNγ levels, which was transient and had no effect on mouse body weight. Toxic inflammatory responses were only observed at high levels of mIL12-F_H_AB (30 µg/mouse), including moderate increases in IL-6, C-reactive protein, and transaminase levels. Overall, a comparison of the PD and toxicological effects of mIL12-F_H_AB in mice suggests that, while the model may be limited to lower doses, B16F10 tumors are well controlled in a dose range that is non-toxic by these measures. Biodistribution studies also suggest delivery to and retention of mIL12-F_H_AB in tumor tissue (25).

An *in vitro* evaluation of SON-1010 using cells from Syrian hamsters, Sprague Dawley rats, beagles, cynomolgus macaques, or humans was tested for albumin binding, potency, and binding of the complex to FcRn; only macaque cells responded physiologically (26). Therefore, macaques have been used for single- and multiple-dose toxicological testing of products based on the F_H_AB platform *in vivo*. After a single SC dose of SON-1010, drug-related changes in clinical observations, body weight, clinical pathology, cytokines, and immunophenotyping were tolerated up to 250 µg/kg in macaques and were consistent with the anticipated on-target effects of rIL-12 (20). Most parameters recovered to pre-study values by day 22, and SON-1010 displayed improved PK characteristics compared to those reported for rIL-12. In the GLP toxicology study, three SC injections of SON-1010 were tolerated in monkeys at up to 62.5 µg/kg/dose. Hematological changes in red blood cells, reticulocytes, platelets, and neutrophils were suggestive of accelerated maturation, along with transient suppression of monocyte, lymphocyte, eosinophil, and white blood cell counts. Minimal changes occurred in the clinical chemistries. Cytokine data showed SON-1010-related effects on IFNγ, with minimal or no changes in IL-6, IL-8, IL-10, IL-1β, or TNFα. The no adverse effect level (NOAEL) in Cynomolgus macaques following repeated SC administration of SON-1010 was defined as 62.5 µg/kg/dose.

The unusual PK results comparing these two clinical studies suggest the potential for an improved local immune response due to accumulation in the TME in patients, which could make SON-1010 more effective than prior efforts with systemic immunotherapy using rIL-12. The dose relationship also suggests TMDD, perhaps due to the retention of SON-1010 caused by albumin binding to SPARC (27) and its slow release from the tumor tissue. Based on the SON-1010 concentration curves, a dose interval of 3 weeks produces minimal accumulation of SON-1010 before the next dose; therefore, any accumulation of the drug is unlikely to be physiologically significant. The drug product used in SB101 was a liquid formulation manufactured in a fed-batch process, while that used in SB102 was lyophilized and had been produced using a perfusion process, which may have accounted for the distinctive PK profiles. However, both drug products passed GMP release and stability testing with nearly identical results, including potency testing using an IL-12 HEK-Blue bioassay that assesses IFNγ production (43). Although subtle differences in biomolecule manufacturing lots are common and can include minor differences caused by deamidation or glycosylation (44), both lots met manufacturing specifications and were considered to be physically and functionally identical. Thus, variations such as these would not be expected to cause the drug elimination profile differences that were observed. Further testing with subsequent doses is required to substantiate the safety of prolonged dosing, which is planned for the next study (SB221).

Overall, the IFNγ PD response with a single dose in SB102 was dose-related, controlled, and prolonged without the stimulation of a more toxic cytokine response (**Figure 6**), which may be required to initiate tumor control in humans, as in mice (45). Neutropenia, lymphopenia, and thrombocytopenia have been reported as common AEs with rIL-12. In the large dose-escalation study of rIL-12 in healthy volunteers (30), dose-related neutropenia reached a nadir on day 5 after a single dose and the mean returned to baseline (or above) by 2 weeks. Neutropenia, lymphopenia, and thrombocytopenia were seen in both SB101 and SB102 with similar nadir and recovery times. The SON-1010 C_max_ can also be compared with the IFNγ response using AUC_0-48h_ in the SB101 cancer patients using a Pearson correlation coefficient (**Supplementary Material Figure S5**). The Pearson correlation coefficient measures linear correlation between two sets of data and is the ratio between the covariance of two variables and the product of their standard deviations; thus, it is essentially a normalized measurement of the covariance, such that the result always has a value between -1 and 1. The Pearson coefficient using C_max_ vs AUC_0-24_ in SB102 was also significant. The longer time to C_max_ may reflect retention in the TME in the cancer patients.

Drugs such as SON-1010, which induce IFNγ in the TME, upregulate the expression of PD-L1 on tumor cells and/or immune cells (46). While there is a reasonable chance that SON-1010 inhibits tumor growth at higher doses, owing to its improved targeting of the TME, SON-1010 may have its greatest effect in treating cancer in combination with an immune checkpoint inhibitor (47). The next development step is to determine the SON-1010 MTD when combined with an immune checkpoint inhibitor in patients with a tumor that is high in SPARC, such as platinum-resistant ovarian cancer, which continues to be a high unmet need indication. Proof-of-concept will be assessed in this population in study SB221, using the combination of SON-1010 with atezolizumab, compared with SON-1010 alone or standard-of-care therapy.

## Data availability statement

The raw data supporting the conclusions of this article will be made available by the authors, without undue reservation.

## Ethics statement

The studies involving humans were approved by Alfred Human Research Ethics Committee and WCG Institutional Review Board. The studies were conducted in accordance with the local legislation and institutional requirements. The participants provided their written informed consent to participate in this study.

## Author contributions

RK: Conceptualization, Data curation, Formal analysis, Investigation, Methodology, Project administration, Software, Supervision, Visualization, Writing – original draft, Writing – review & editing. JC: Conceptualization, Methodology, Project administration, Visualization, Writing – review & editing. SD: Conceptualization, Methodology, Project administration, Writing – review & editing. MD: Investigation, Project administration, Supervision, Writing – review & editing. JB: Conceptualization, Data curation, Formal analysis, Methodology, Software, Supervision, Validation, Visualization, Writing – review & editing. IK: Data curation, Formal analysis, Investigation, Validation, Visualization, Writing – review & editing. SC: Conceptualization, Investigation, Methodology, Supervision, Writing – review & editing. TP: Supervision, Writing – review & editing, Investigation, Methodology, Project administration. JL: Investigation, Methodology, Project administration, Supervision, Writing – review & editing. PR: Investigation, Methodology, Project administration, Supervision, Writing – review & editing.
