# Supplementary material for: A phase I trial of SON-1010, a tumor-targeted, interleukin-12-linked, albumin-binding cytokine, shows favorable pharmacokinetics, pharmacodynamics, and safety in healthy volunteers
Source: Front Immunol. 2024 Feb 29;15:1362775. doi: 10.3389/fimmu.2024.1362775 (PMC10937388; doi:10.3389/fimmu.2024.1362775)
Supplement: Supplementary file 1 [file DataSheet_1.pdf]

Supplementary Material

1 SB102 Supplementary Information

1.1 Inclusion Criteria

Participants are eligible to be included in the study if all the following criteria apply:

1. Participants must be medically healthy based on medical history, physical examination, and clinical laboratory testing.

At the time of the informed consent:

2. Participants must be between 18 to 54 years of age, inclusive.

3. May only be limited users of nicotine-containing products, including e-cigarettes, for at least 3 continuous months before the first dose of SON-1010, as confirmed by cotinine testing at screening and check-in (day -1). Social smoking is allowed up to 5 cigarettes per week as long as the participant is willing to abstain during confinement and the cotinine test is negative at check in. Repeat testing is permitted at screening.

4. Must have negative drug/alcohol testing at screening and check-in (day -1). Screening drug/alcohol testing may be repeated if deemed appropriate by the Principal Investigator.

5. Must have vital signs (after sitting for at least 5 minutes) within the following ranges at screening and check-in (day -1):
   a. Systolic blood pressure (BP), 90 to 140 mmHg, inclusive
   b. Diastolic BP, 40 to 90 mmHg, inclusive
   c. Heart rate (HR) >50 to ≤100 beats per minute

6. Must weigh >50 kg and ≤100 kg and have body mass index (BMI) ≥18 and ≤32 kg/m^2 at screening.

7. Must have normal laboratory function by day -1 (note that the screening safety laboratory assessment may be repeated to confirm the initial result and trending), defined as:
   a. Creatinine level ≤ upper limit of normal (ULN) for age
   b. Alanine aminotransferase (ALT) ≤ ULN
   c. Total bilirubin ≤ ULN (unless participant has Grade 1 bilirubin elevation due to Gilbert's disease or a similar syndrome involving slow conjugation of bilirubin and direct bilirubin is <35%)
   d. Albumin > lower limit of normal (LLN)
   e. Coagulation International Normalized Ratio (INR) < 1.5

8. Females of childbearing potential, <1-year postmenopause who are not permanently sterile, must have a negative pregnancy test (beta-human chorionic gonadotropin [β-HCG]) at

day -1, and agree to abstinence or use 2 highly effective methods of birth control for 30 days before the study, during the study, and for 30 days after the last dose of study intervention. Females who are not of childbearing potential (have had a hysterectomy, bilateral oophorectomy, or are ≥ 1-year postmenopause) do not need to use any contraception.

Nonchildbearing potential is defined as surgically sterile or postmenopausal (defined as 12 months of spontaneous amenorrhea). A follicle stimulating hormone (FSH) level >40 IU/L at screening will confirm postmenopausal status. If a participant is not sexually active, but becomes active, then she and her male partner must use 2 methods of adequate contraception.

9. Males and their female partners must use a highly effective method of birth control if female partner(s) is of childbearing potential and must not donate sperm during the study and for 90 days after the last dose of study intervention.

10. Willing and able to provide signed informed consent, which includes compliance with the requirements and restrictions listed in the informed consent form (ICF) and in this protocol.

11. Must be able to communicate well with Investigator and/or study site personnel and to comply with the requirements of the entire study.

1.2 Exclusion Criteria

Participants are excluded from the study if any of the following criteria apply:

1. Concurrent conditions that could interfere with safety and/or tolerability measurements.

2. Known history of allergy to any component of study intervention.

3. History of severe allergic/anaphylactic reaction.

4. Pregnancy and/or lactation

5. Receipt of any plasma-, protein-, or antibody-based therapeutic agents (e.g., growth hormones or monoclonal antibodies) within 3 months before the first dose of study intervention.
   Note: Influenza and COVID-19 vaccines will be allowed if administered more than 14 days before the first dose of study intervention.

6. Receipt of any investigational agent or treatment within 30 days or 5 half-lives, whichever is longer, before the first dose of study intervention.

7. Any active infection, including COVID-19, as determined by the currently applicable standard before study intervention.

8. Any acute noninfectious illness within 30 days before day 1.

9. Diagnosis of or positive screening result for active COVID-19, hepatitis B surface antigen (HbsAg), hepatitis C virus antibody (HCVAb), or human immunodeficiency virus (HIV)-1 or HIV-2 antibody.

10. Unable or unwilling to cooperate with the Investigator for any reason.

11. History of any clinically relevant or chronic psychiatric, renal, hepatic, cardiovascular, pancreatic, neurologic, hematologic, or gastrointestinal disease (e.g., inflammatory bowel disease) or current clinically significant liver function test results. Mild anxiety and depression may be acceptable at the investigator's discretion.

12. Evidence of any surgical or medical condition that, in the judgment of the Investigator, might interfere with the absorption, distribution, metabolism, or excretion of study intervention. History of cholecystectomy is acceptable.

13. Presence of clinically significant electrocardiogram (ECG) finding (confirmed upon repeat testing) that may interfere with any aspect of study conduct or interpretation of results, as follows:
    a. QT interval corrected for HR according to Fridericia's formula (QTcF) > 450 in males or > 470 msec in females at screening or check-in (day -1).
    b. Other ECG abnormalities clinically relevant in the judgment of the Investigator.

14. Use of any prescription or over-the-counter medications (apart from standard doses of analgesics), herbal products (e.g., St. John's Wort, garlic supplements, milk thistle), topical or inhaled steroids, or supplements/vitamins within 7 days before the first dose of SON-1010 and for the duration of the study (oral contraceptives, hormone replacement therapy, or other products that would not interfere with the safety or immune response are acceptable).

15. Recent use of systemic steroids within 14 days of enrollment, except for physiologic doses for steroid replacement. Limited doses of systemic steroids (e.g., in patients with exacerbation of reactive airway disease) must have been completed at least 10 days before enrollment.

16. Use of biotin (i.e. Vitamin B7) or supplemental biotin higher than the daily adequate intake of 30 µg. Patients who switch from a high dose to a dose of 30 µg/day or less are eligible.

17. History of alcohol or substance abuse within 6 months before the first dose of SON-1010 per the criteria in the Diagnostic and Statistical Manual of Mental Disorders, Fifth Edition (DSM-V).

18. History of any major surgery within 3 months before the first dose of study intervention.

19. Donation or receipt of blood or blood product within 3 months before the first dose of SON-1010 and for the duration of the study.

Figure S1: Flow Diagram for SB102

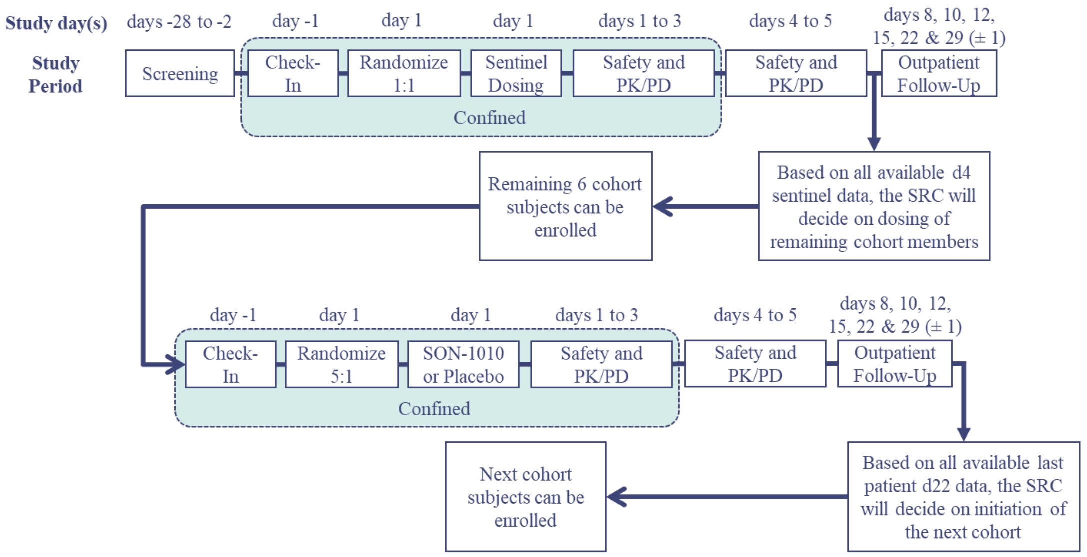

Sentinel participants were screened, then confined for the first three days after blinded dosing for close observation and subsequently followed in the clinic. Safety was reviewed by the SRC after the day 4 visit before approving enrollment of the rest of each cohort and the process was repeated. Once all subjects in each cohort had been followed for 22 days, their safety was reviewed by the SRC before approving the next dose cohort.

2 SB102 Supplementary Data

Figure S2: SB102 Representative Acute Inflammatory Cytokine Results

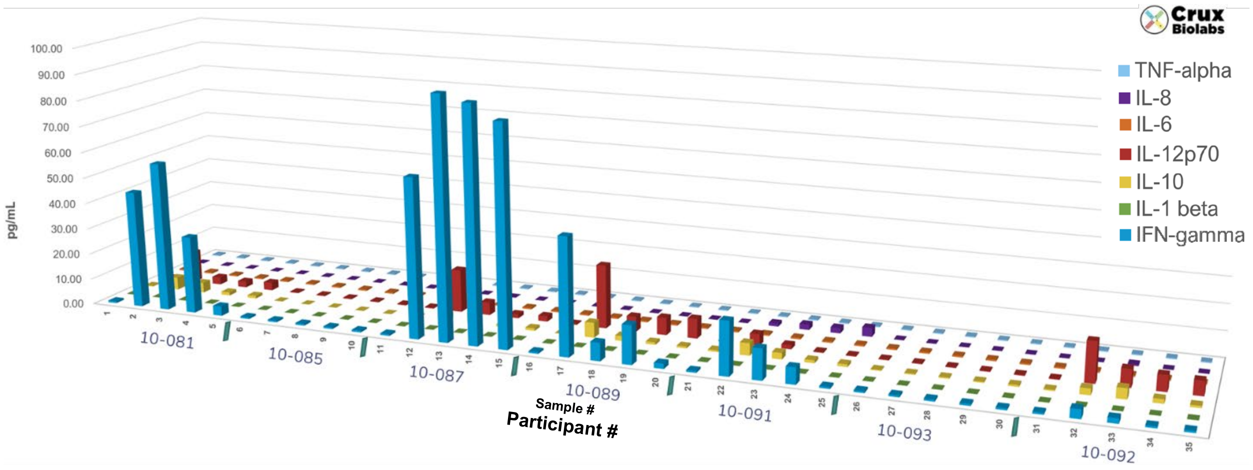

Acute Inflammation parameters including IFNγ, IL-1β, IL-6, IL-8, IL-10, IL-12p70, and TNFα, were assayed at Crux using the Luminex platform. Results are shown from the final dose cohort (S4) as days 1, 2, 3, 5, and 8 (for each consecutive sample number) after the SC dose for each participant. SON-1010 (as measured by IL-12p70) levels peaked at 24-48 hours. Increases in IFNγ (showing an IL-12 effect and potential for tumor control) were dose-related, controlled, and prolonged. Low amounts of IL-10 were induced with each dose in a dose-dependent manner, which could also be a result of the increase in IFNγ. No consistent pattern of response was seen with IL-1β, IL-6, IL-8, or TNFα and there was no evidence of cytokine release syndrome (CRS).

3 SB101 vs. SB102 Comparisons

Figure S3: SON-1010 PK Parameters by Treatment Comparing SB102 with SB101

A: SB102 in Healthy Volunteers

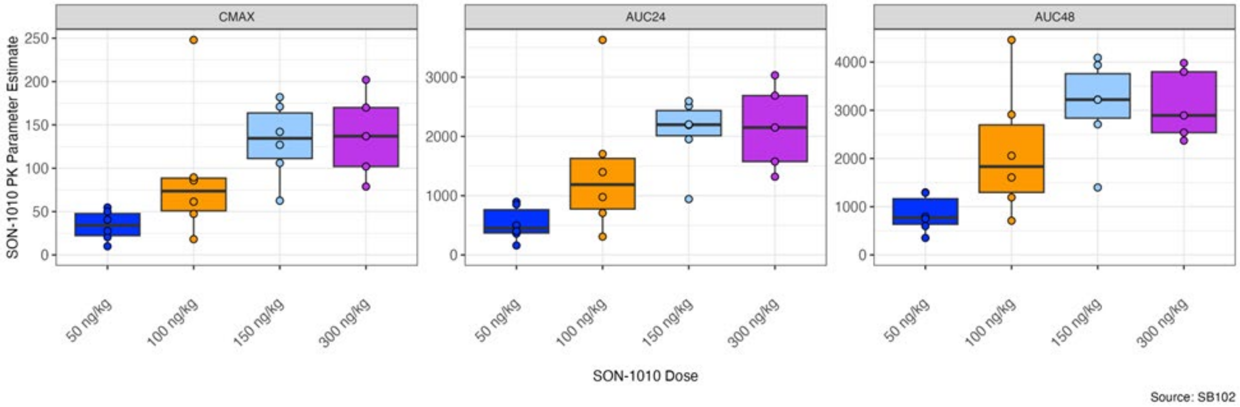

B: SB101 in Cancer Patients

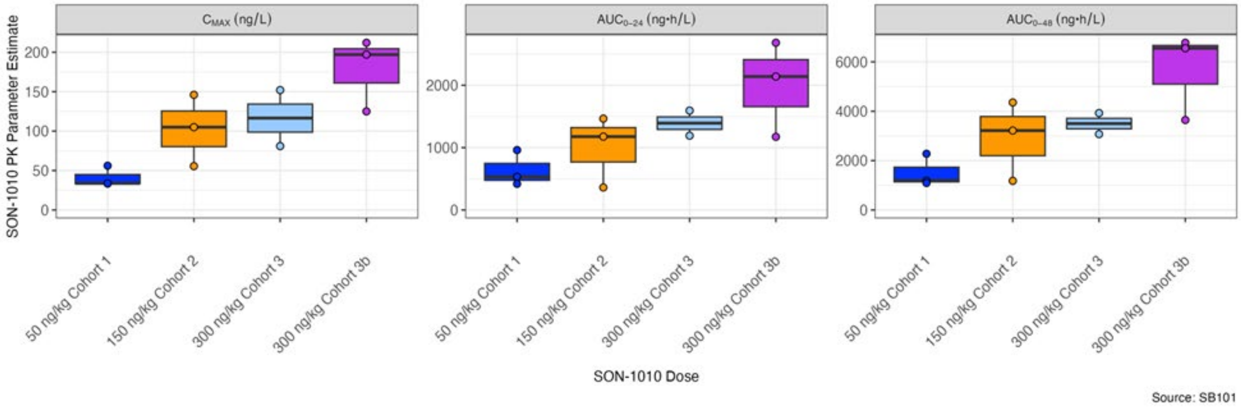

(A) Serum PK was monitored over time in the SB102 healthy volunteers after a single SC dose of SON-1010 as indicated for each group. The C_{max} (pg/mL), AUC_{0-24} (h•pg/mL), and AUC_{0-48} (h•pg/mL) PK parameters are shown graphically as box plots to display the median, lower, and upper quartiles, as well as the lower and upper extremes. (B) Serum PK was also monitored over time in the SB101 cancer patients; results after SC injection of the maintenance (2nd) dose of SON-1010 are displayed. Identical PK parameters are shown for comparison.

Figure S4: PD Parameters by Treatment Comparing SB102 with SB101

A: SB102 IFNγ PD Response in Healthy Volunteers

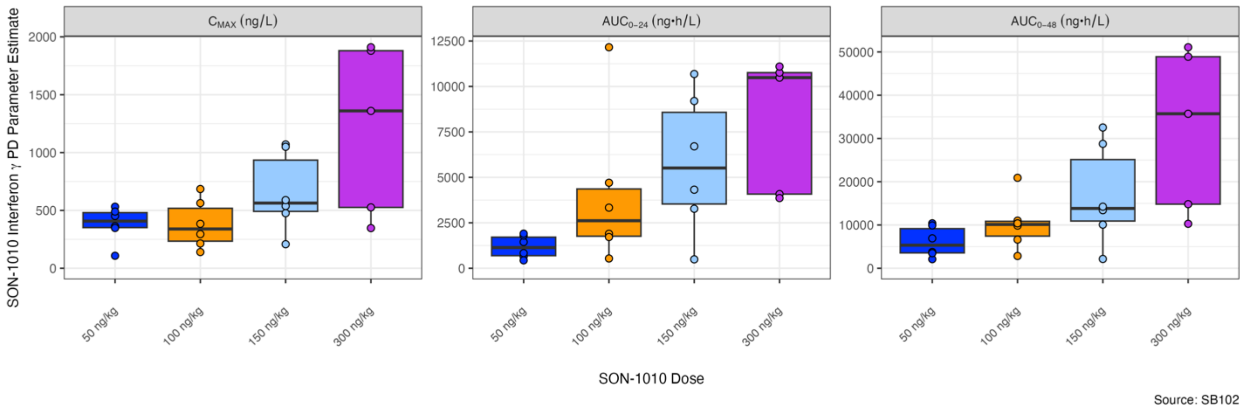

B: SB101 IFNγ PD Response in Patients with Cancer

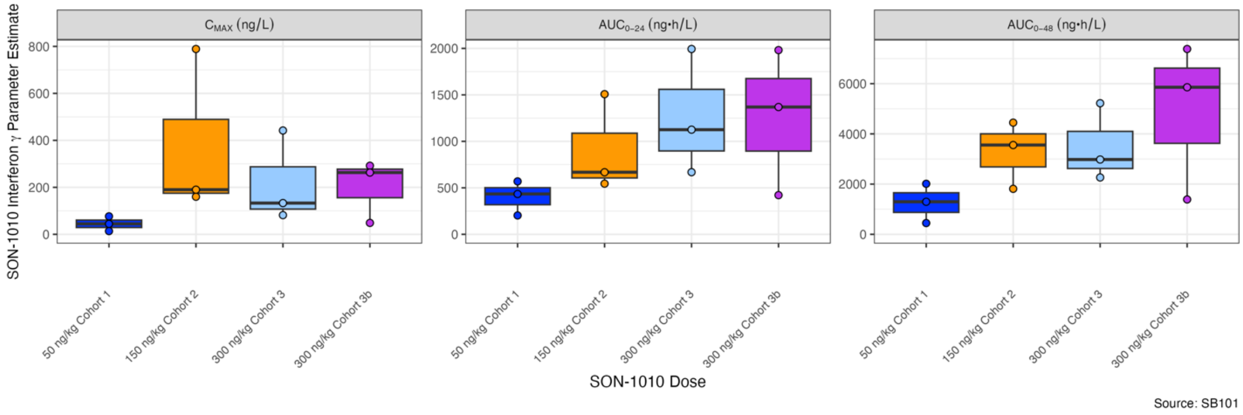

(A) Serum IFNγ was monitored over time in the SB102 healthy volunteers after a single SC dose of SON-1010 as indicated for each group. The C_{max} (pg/mL), $AUC_{0\text{-}24}$ (h•pg/mL), and $AUC_{0\text{-}48}$ (h•pg/mL) PK parameters are shown graphically as box plots to display the median, lower and upper quartiles, and lower and upper extremes. (B) Serum IFNγ was also monitored over time in the SB101 cancer patients; results after SC injection of the maintenance (2nd) dose of SON-1010 are shown. Identical PK parameters are shown for comparison.

Figure S5: Pearson Correlation Coefficients Evaluate PD Effect

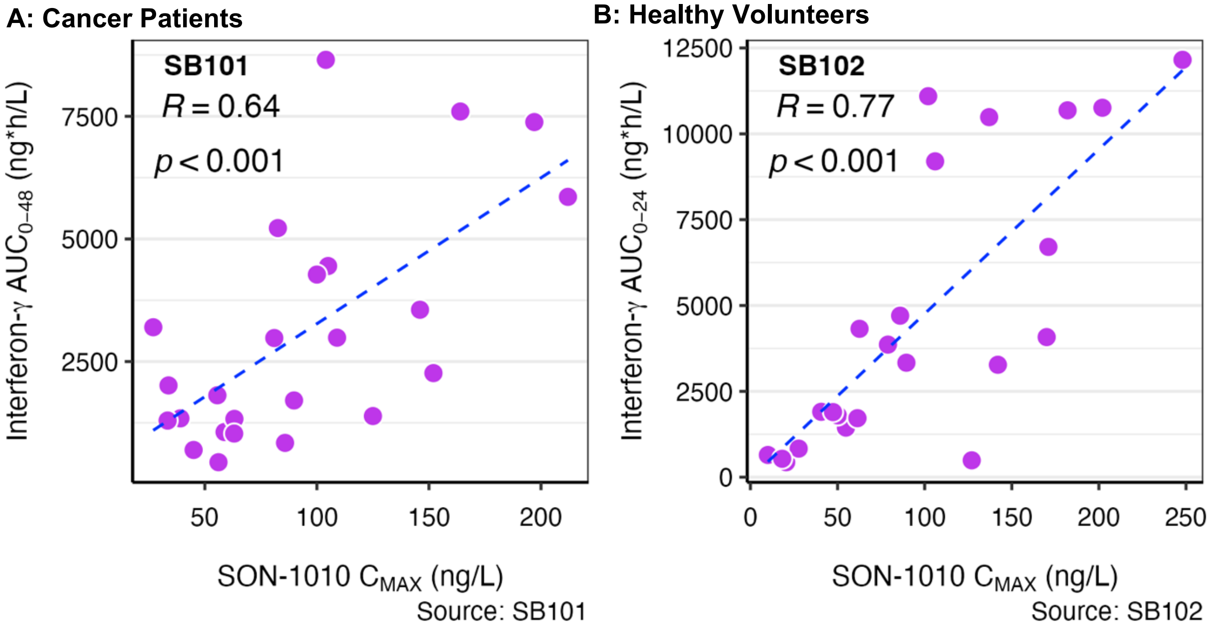

(A) Each PK or PD parameter was compared with the others in the SB101 cancer patients who had received their maintenance (2nd) dose of SON-1010 and a coefficient (R) was determined. The correlation between the SON-1010 C_{max} with IFNγ AUC_{0-48} shows the impact of the IL-12 on the IFNγ response. (B) Similar comparisons (at C_{max} after 24 hours) are shown for the SB102 parameters.
